# Metabarcoding of fecal pellets in wild muskox populations reveals negative relationships between microbiome and diet alpha diversity

**DOI:** 10.1002/ece3.10192

**Published:** 2023-06-13

**Authors:** Erin Prewer, Sibelle T. Vilaça, Samantha Bird, Susan Kutz, Lisa‐Marie Leclerc, Christopher J. Kyle

**Affiliations:** ^1^ Environmental and Life Sciences Graduate Program Trent University Peterborough Ontario Canada; ^2^ Forensic Science Department Trent University Peterborough Ontario Canada; ^3^ Department of Ecosystem and Public Health, Faculty of Veterinary Medicine University of Calgary Calgary Alberta Canada; ^4^ Department of Environment Government of Nunavut Kugluktuk Nunavut Canada; ^5^ Natural Resources DNA Profiling and Forensic Centre Peterborough Ontario Canada

**Keywords:** alpha diversity, beta diversity, diet, gut microbiome, metabarcoding, muskox, *Ovibos moschatus*

## Abstract

Microbiome diversity and diet composition concomitantly influence species health, fitness, immunity, and digestion. In environments where diet varies spatially and temporally, microbiome plasticity may promote rapid host adaptation to available resources. For northern ungulates in particular, metabarcoding of noninvasively collected fecal pellets presents unprecedented insights into their diverse ecological requirements and niches by clarifying the interrelationships of microbiomes, key to deriving nutrients, in context of altered forage availability in changing climates. Muskoxen (*Ovibos moschatus*) are Arctic‐adapted species that experience fluctuating qualities and quantities of vegetation. Geography and seasonality have been noted to influence microbiome composition and diversity in muskoxen, yet it is unclear how their microbiomes intersect with diet. Following observations from other species, we hypothesized increasing diet diversity would result in higher microbiome diversity in muskoxen. We assessed diet composition in muskoxen using three common plant metabarcoding markers and explored correlations with microbiome data. Patterns of dietary diversity and composition were not fully concordant among the markers used, yet all reflected the primary consumption of willows and sedges. Individuals with similar diets had more similar microbiomes, yet in contrast to most literature, yielded negative relationships between microbiome and diet alpha diversity. This negative correlation may reflect the unique capacities of muskoxen to survive solely on high‐fiber Arctic forage and provide insight into their resiliency to exploit changing dietary resources in a rapidly warming Arctic altering vegetation diversity.

## Graphical abstract

We compared microbiome and diet diversity in muskoxen using metabarcoding of three common plant markers. Our results show that individuals with similar diets had more similar microbiomes yet yielded negative relationships between microbiome alpha diversity and diet.

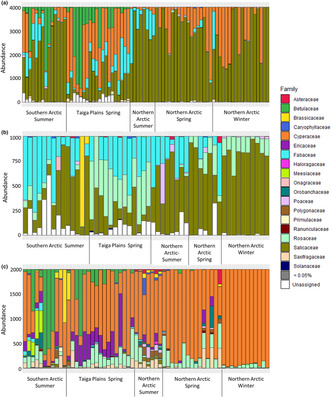

## 1 INTRODUCTION

Climate change has dramatically changed both terrestrial and aquatic ecosystems, affecting the ecology and evolution of many organisms and communities (Espunyes et al., 2022; Hoffmann et al., 2019). Increasing global temperatures have resulted in changing and novel selective pressures for many species, which can lead to population declines, local extirpations, and overall lowered biodiversity (Bright Ross et al., 2021; Hamann et al., 2021; Lovari et al., 2020). Vegetation is particularly sensitive to changes in precipitation and temperature, where climate change has been associated with decreased nutrient availability, droughts, increased pests, soil erosion, and increased competition (Akram et al., 2022; Bright Ross et al., 2021; Elbasiouny et al., 2022; Espunyes et al., 2022; Hamann et al., 2021). Reduced plant productivity and quality, as well as altered plant phenology, have a cascading negative effect on herbivore species that depend on these resources (Espunyes et al., 2022; Hamann et al., 2021; Lovari et al., 2020). For example, environmental warming is expected to reduce the availability of snow bed vegetation, an important food source for Apennine chamois (*Rupicapra pyrenaica*), and is thus predicted to increase juvenile mortalities, potentially leading to local extirpations (Lovari et al., 2020). While some alpine herbivore species, like the ibex (*Capra ibex*) or alpine chamois (*Rupicapra rupicapra*) can adjust to changing vegetation availability via behavioral changes (e.g., foraging bite and step rates) or range shifts to higher elevations (Espunyes et al., 2022; Lovari et al., 2020), these changes are thought to compromise fitness (Bright Ross et al., 2021). Alternatively, some species have the capacity to adapt to environmental modifications brought about by climate change (Fulgione & Buglione, 2022). For example, beech (*Fagus sylvatica*) yields have increased in Sweden; an important food source for wild boars (*Sus scrofa*; Fulgione & Buglione, 2022), where additional food availability is expected to result in population growth given increases in lifespan, earlier female sexual maturity, and decreased piglet mortality rates (Fulgione & Buglione, 2022). As outcomes of climate change vary, there is a demonstrated need to better understand the capacity of affected species to adapt to rapid environmental changes.

Gut microbiome compositions are increasingly recognized as contributors to the ability of species to respond to rapidly changing environments by combating pathogens, expanding dietary niches through digestion and individual metabolic function, and enhancing heat tolerance (Bolnick et al., 2014; Buglione et al., 2022; Hicks et al., 2018; Li et al., 2016; Loo et al., 2019; Rothschild et al., 2018; Wu et al., 2018). It is not always clear, however, how microbiome diversity and composition are impacted by changing diet in influencing individual health, fitness, nutritional status, and even population dynamics (Kartzinel et al., 2019; Zeineldin et al., 2018). The relationships between the microbiome and diet are especially important in herbivore species as microbiome bacteria (commonly *Bacteroidetes* and *Firmicutes* species) help extract nutrients from plant materials (that can otherwise be unsustainable or nutrient‐poor) and break down toxic plant compounds (Bergmann et al., 2015; Dearing & Kohl, 2017; Ungerfeld et al., 2018). Further, extended fermentation by bacteria in ruminants increases the production of short‐chain fatty acids; metabolic end products absorbed by ruminants to help fulfill energy requirements that are also important signaling molecules with antimicrobial and anti‐inflammatory properties that regulate energy metabolism and macromolecule synthesis (Andersen‐Ranberg et al., 2018; Shabat et al., 2016). By contrast, altered or imbalanced microbiomes can lead to both decreased digestion and gastrointestinal disease that threaten nutritional status and survival (Belanche et al., 2021). In cows, high energy feed containing increased levels of starch or carbohydrates can result in dysbiosis, or the imbalance of bacteria, which negatively affects gastrointestinal tract absorption capacity (Neubauer et al., 2020). In natural systems, metabarcoding of American Bison (*Bison bison*) diet from fecal samples found *Tenericutes* bacteria increased in abundance in response to the seasonal availability of high caloric and high protein content plants (Bergmann et al., 2015). Thus, the plasticity of the microbiome in responding to external biotic and abiotic factors (e.g., season, geography, disease, and diet), may allow individuals to adjust to their environment and exploit different ecological niches (Loo et al., 2019; Ng et al., 2018; Reese & Dunn, 2018). The extent and effect that diet has on microbiome composition varies greatly by species, as do the physiological effects that microbiome plasticity can have on individuals; thus impacting their capacity to acclimate to their environment (Diaz & Reese, 2021). The culmination of the aforementioned studies suggests health, fitness, and sustainability assessments of wild populations would be further enhanced through a better understanding of the seasonal and geographical dietary factors that influence microbiome compositions and diversity in natural systems.

The Arctic is an extreme environment characterized by temperatures regularly ranging from −35 to 15°C, extreme photoperiods, and severe weather events leading to reduced vegetation biodiversity throughout the year (Berger et al., 2018; Blix, 2016; Callaghan et al., 2004). The Arctic is warming at nearly four times the global rate, reducing plant diversity, shifting plant dispersal patterns, and altering plant phenology (Ernakovich et al., 2014; Olofsson et al., 2009; Speed et al., 2021); drastic changes threatening the sustainability of Arctic herbivores. Muskoxen (*Ovibos moschatus*) are large Arctic ruminants whose range encompasses the Arctic archipelago, Greenland, Russia, and Alaska (Cuyler et al., 2019), and are culturally, economically, and nutritionally significant to the Indigenous Peoples around the Arctic (Prewer et al., 2022; Tomaselli et al., 2018). Muskoxen are keystone species that contribute to ecosystem health via soil nutrient turnover (Mosbacher, Michelsen, et al., 2016). While muskoxen are currently considered species of least concern by the International Union for Conservation of Nature (IUCN) given their current global population size, there are fears that the long‐term sustainability of this iconic Arctic species is threatened by continued, rapid environmental warming (Cuyler et al., 2019; Gunn & Forchhammer, 2022). These concerns are substantiated by population collapses on Victoria and Banks Island, which previously held 61% of Canada's overall muskox population (Cuyler et al., 2019; Gunn & Forchhammer, 2022; Kutz et al., 2015). Muskoxen are long‐lived species with notoriously low levels of genetic diversity, characteristics predicted to reduce their capacity to adapt on timescales reflective of rapidly changing Arctic environments (Hansen et al., 2018; Prewer et al., 2019). As such, other adaptive mechanisms such as microbiome plasticity may be key to the long‐term survival of muskoxen (Bird et al., 2019; Hansen et al., 2018; Prewer et al., 2019).

Muskox microbiomes consist mainly of *Firmicutes* and *Bacteroidetes* that digest carbohydrates and fiber, consistent with observations in other ruminant species like cattle, bison, goats, and sheep (Bird et al., 2019). The ability of muskoxen to digest a high‐fiber diet is key to their survival in the Arctic as it allows them to eat vegetation that other Artic herbivores, like caribou and hares, avoid given their low‐nutrient status (Bird et al., 2019; Salgado‐Flores et al., 2016; Schmidt et al., 2018). Variations in wild muskox microbiomes have been associated with environmental and host factors such as body mass and population of origin (Andersen‐Ranberg et al., 2018; Bird et al., 2019). Additionally, Bird et al. (2019) found geography and seasonality had strong effects on abundance, diversity, and richness of bacterial species in muskoxen, with higher bacterial diversity observed in more northern regions and winter months (Bird et al., 2019). While the diversity and abundance of ingested vegetation in muskoxen are thought to have concomitant effects on the microbiome, this hypothesis has not been directly tested in wild populations.

**FIGURE 1:**
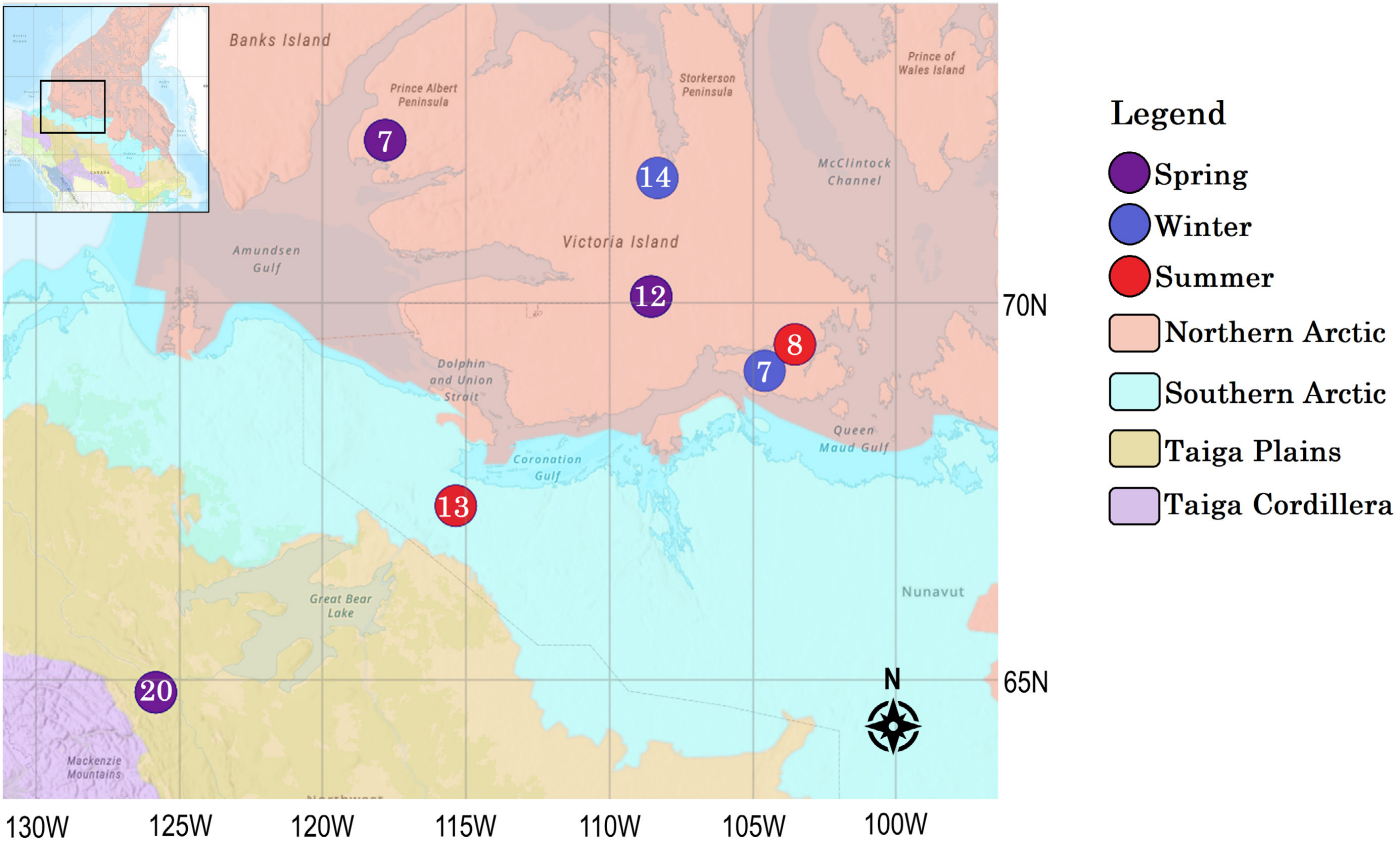
Map of sampling sites included in this study. Ecozones are differentiated by base map color (ESRI Canada); season of collection is indicated via circle color; and the number of samples collected per sampling location is indicated by the number inside circle.

Muskoxen are grazers, where previous studies suggest they have a narrow dietary niche made up mostly of graminoids, sedges, and willows, all of which are considered low‐quality forage (Adamczewski, Flood, et al., 1994; Adamczewski, Kerr, et al., 1994; Schmidt et al., 2018; Staaland et al., 1997; Ungerfeld et al., 2018). In Canada, muskoxen span a wide latitudinal range from the mainland of Nunavut and Northwest Territories to the high Arctic islands, consisting of several ecozones that vary in vegetation types and abundances (Figure 1). This variation, as well as clinal changes in vegetation morphology and nutritional status, is thought to result in latitudinal shifts in diet, especially between mainland and island populations (Smith et al., 2002). Muskox diet has also been found to vary seasonally, as access to vegetation is restricted during winter, where icing and snowfall reduces the quantity of food available (Ihl & Klein, 2001; Larter & Nagy, 1997, 2004; Mosbacher, Michelsen, et al., 2016). Ihl and Klein (2001) found that while muskoxen select for graminoids, suitable snow conditions led them to feed predominantly in high lichen cover areas with their feces containing high occurrences of lichen. As climate change impacts Arctic forage abundance and distributions, it is unclear whether vegetation changes will influence muskox gut bacteria and undermine fitness. Direct comparisons of muskox microbiome and diet diversity may help in understanding how muskoxen interact with their environments and the influence climate change may have on their health.

Previous descriptions of muskox diet variability have been mostly based on broad vegetation indexes, microhistology, and stable isotope analyses. While informative, these approaches are limited in resolving finer‐scale changes in diet (Ihl & Klein, 2001; Larter & Nagy, 1997, 2004; Mosbacher, Michelsen, et al., 2016). Metabarcoding allows for species identification in mixed samples through high throughput sequencing (Bush et al., 2019; Ruppert et al., 2019; Zepeda Mendoza et al., 2015), where a variety of genetic markers are used to inform a myriad of applications such as ecosystem biomonitoring, environmental responses to pollution, and the characterization of the gut microbiome (Bird et al., 2019; Ruppert et al., 2019). DNA metabarcoding of plant markers has also been established as a highly sensitive, accurate, and noninvasive method to assess diet, particularly from fecal samples (Boukhdoud et al., 2021). Diet metabarcoding was performed on 20 muskoxen from Greenland by Schmidt et al. (2018), where their diet was found to consist mainly of forbs and graminoids, consistent with previous muskox diet analyses. The validation of diet analyses via fecal plant metabarcoding in muskoxen therefore allows for the integration of microbial and vegetation metabarcoding data, with the potential to better describe and elucidate relationships between muskox diet and microbiome diversity.

In this study, we amplify three common plant markers, the P6 loop of the chloroplast trnL (UAA) intron region (TRNL), ribulose 1,5‐biphosphate carboxylase region (RBCL), and Internal Transcribed Spacer region (ITS) from fecal samples to assess the diet of wild muskoxen across much of their Canadian range. We compared the diversity and composition of the diet to that of the microbiome to determine how vegetation in different landscapes contributes to bacterial variation in muskox gut microbiomes. Data provided herein offer insight into the plasticity of microbiomes relative to dietary composition, which in turn also allow for predictions of the long‐term impact climate change and concomitant vegetation changes have on muskox viability.

## 2 METHODS

### 2.1 Plant DNA analysis

Fecal pellet samples and DNA used in this study were previously selected and extracted by Bird et al. (2019) as part of a gut microbiome investigation of 78 wild male muskoxen. These samples were collected from six regions, encompassing two genetically distinct populations (Victoria Island and mainland individuals), three ecozones (taiga plains, southern Arctic, and northern Arctic), and three seasons (spring, summer, and winter) (Figure 1). The extracted DNA from these 78 samples was used herein for plant metabarcoding in order to directly compare microbiome and vegetation diversity.

We employed three primer pairs commonly used for plant metabarcoding including chloroplast TRNL region using the g/h primer set from Taberlet et al. (2007); chloroplast RBCL region using the z1aF/hp2R primer set from Hofreiter et al. (2000); and an Internal Transcribed Spacer region, ITS, using 2F/3R primers from Chen et al. (2010). These three markers were chosen as they sequenced both chloroplast and nuclear DNA and had different amplicon lengths, which provided the ability to amplify degraded DNA while still retaining the sequence diversity to differentiate between species. Amplicon lengths were ~150, 300, and 500 bp, respectively. Genes were amplified and libraries were prepared using a modified version of the 16s metagenomic Illumina protocol (Illumina). Total amplification reaction volumes were halved using 6.25 μL of Kappa HiFi mastermix (Roche), 0.38 μL of 10 μM forward and reverse primer, 3.5 μL of water, and 2 μL of stock extracted DNA for a total volume of 12.5 μL per sample. Amplification temperatures and cycle numbers were optimized on pure soy DNA as a control, as well as a subset of muskox fecal samples using temperature gradients. TRNL and RBCL were amplified at 62°C, and ITS was amplified at 60°C with 35 cycles to amplify all markers. All muskox samples were amplified in triplicate and visualized on an ethidium bromide‐stained 1.5% agarose gel along with negative controls of ultrapure water and positive controls consisting of soy DNA. Triplicate samples were pooled, cleaned, and size‐selected using AMPure XP beads (Beckman Coulter Life Sciences) with a bead ratio of 1.2 for TRNL and 0.8 for RBCL and ITS. Samples were indexed for sequencing using dual 8 bp combinations following the Illumina 16s metabarcoding protocol where volumes were once again halved for a total of 25 μL. Following a final AMPure XP bead clean up, Quant‐IT dsDNA Picogreen kit (ThermoFisher) was used on a FLUOstar Omega microplate reader to determine the DNA concentration of each sample for each marker. Samples were normalized to a standard concentration per marker and pooled to obtain a final library for each marker. Libraries were then sent to the University of Guelph's AAC genomics facility for sequencing on an Illumina MiSeq using a 600‐cycle V3 kit to produce 2 × 300 bp reads.

### 2.2 Plant library filtering

Data were analyzed using the Quantitative Insights into Microbial Ecology 2 (QIIME2) pipeline v.2019.7 (Bolyen et al., 2019). QIIME2 was first used to demultiplex samples after which primer and adapter sequences were trimmed using cutadapt wrapper followed by denoising performed using the dada2 algorithm. A reference database of TRNL sequences was created from sequences uploaded to the NCBI database. Sequences were then filtered to remove duplicates and only contain plant and fungi nucleotide sequences from the respective chloroplast regions. Taxon IDs were extracted from sequences to create a corresponding lineage database for each taxon. The final database contained 288,520 sequences from 161,845 taxa. For RBCL, full data records were downloaded from the Barcode of life database (BOLD), which included taxonomy and nucleotide sequences for all plant species. The sequences were filtered to only contain sequences that were from RBCL markers and had taxonomy information down to the family level at minimum. The taxonomy and sequence information was then extracted and filtered further to remove duplicates and low‐quality sequences. This resulted in 128,125 sequences of 47,665 taxa. A high‐quality QIIME2 formatted database created by Banchi et al. (2020) was used as a reference for the ITS marker using the ITS2 dataset. Sequences for the RBCL and TRNL markers were then compared with their respective databases to create amplicon sequence variants (ASVs) that were taxonomically classified using BLAST+. ITS sequences were taxonomically classified using the sklearn algorithm. Libraries from each marker were then rarified for sequencing depths of 2000 for RBCL, 4000 for TRNL, and 1000 for ITS.

### 2.3 Plant data analysis

Rarefied and nonrarefied datasets were imported into R (v4.2.1; R Core Team, 2022) for statistical analyses. First, phyloseq (v1.40.0; McMurdie & Holmes, 2013) was used to create relative abundance bar plots for all three markers using the rarefied dataset. Beta diversity correlation between ASVs and Shannon index of TRNL, RBCL, ITS, and the microbiome were compared using Bray–Curtis dissimilarity and Jaccard dissimilarity using Spearman correlations with the Vegan package (Oksanen et al., 2020). Rarefied data was used for these analyses to allow for sample and marker comparisons where sample size and sampling depth varied (Cameron et al., 2021). Nonrarefied data from the three diet markers, as well as from Bird et al. (2019), was then used to compare alpha diversity between the diet and microbiome observed ASVs and Shannon index using Spearman's rank correlation using Vegan v.2.5‐7 (Oksanen et al., 2020). The nonrarefied dataset was used to calculate alpha diversity statistics to reduce the risk of bias and provide a more thorough representation of diversity by detecting all differentially abundant ASVs (Bird et al., 2019; McMurdie & Holmes, 2013). Correlation was measured between ASVs of TRNL and microbiome, RBCL and microbiome, and ITS and microbiome and repeated with the Shannon indexes of these samples. Additionally, these comparisons were made between TRNL, RBCL, and ITS markers to determine how similar data were between the three markers.

## 3 RESULTS

### 3.1 Sequencing and library analysis

A total of 4,882,634 paired reads were sequenced for TRNL, ranging from 223 to 227,408 paired reads per individual. RBCL had a total of 1,682,655 paired reads sequenced, ranging from 22 to 93,399 paired reads per individual. ITS had a total of 882,042 paired reads ranging from 8 to 92,073 per individual. Denoising left 2,109,690 (43%) sequences for TRNL, with an average of 25,418 reads per individual, RBCL retained 1,335,114 (82%) sequences with an average of 16,689 reads per individual and ITS had 186,475 (21%) sequences left with an average of 2330 sequences per individual. Remaining TRNL reads were clustered into 227 Amplicon Sequence Variants (ASVs), where RBCL clustered into 188 ASVs, and ITS reads were clustered into 212 ASVs. The final rarefied dataset had an equal sequencing depth of 4000 reads for TRNL, 2000 reads for RBCL and 1000 reads for ITS.

### 3.2 Muskox diet

**FIGURE 2:**
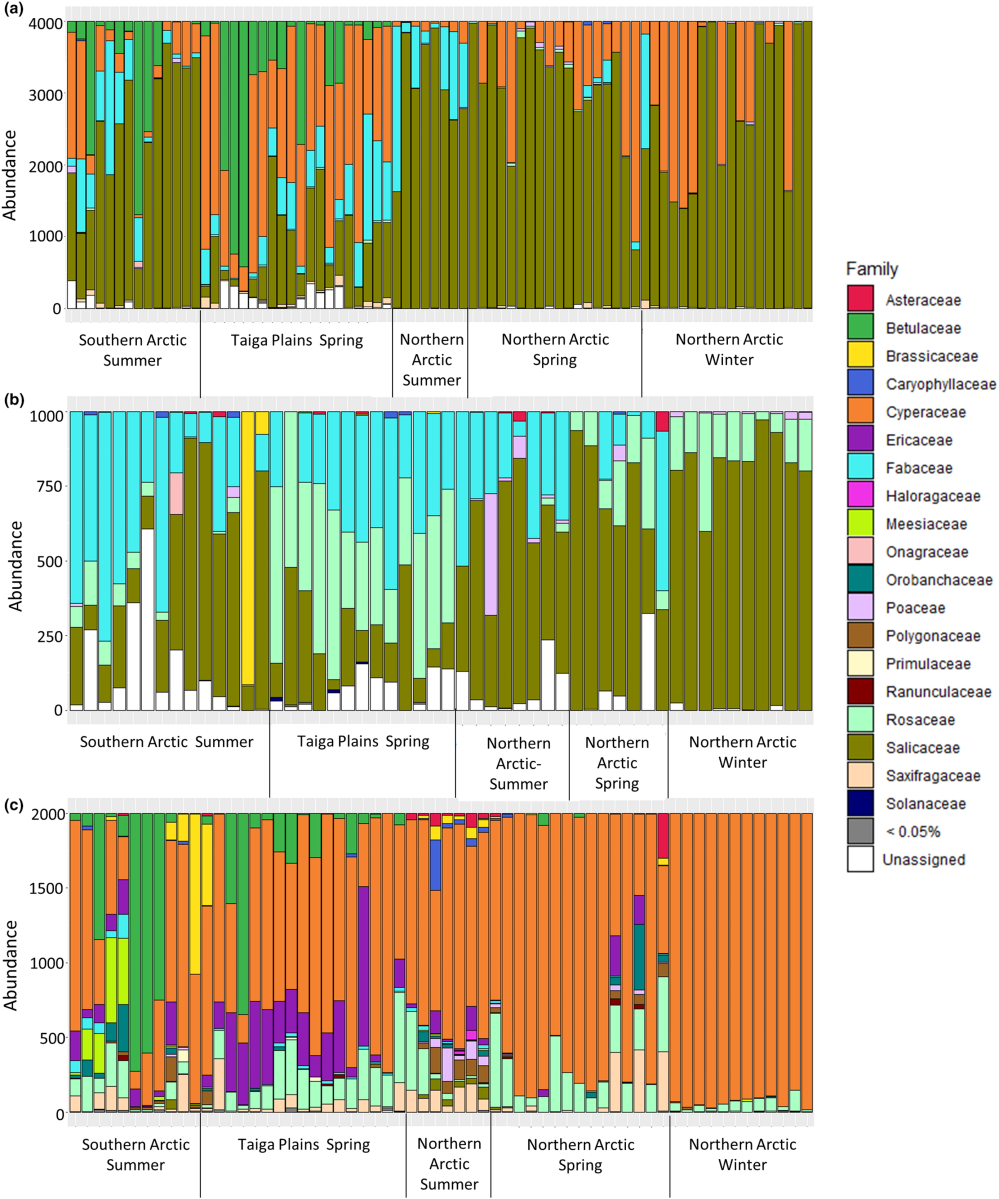
Taxonomic abundance plot for the top plant families from: (a) TRNL, (b) ITS, and (c) RBCL markers classified in QIIME2. Each bar represents an individual with colors showing the ratio of each family found in the reads. Plant families that made up under 0.05% of the overall reads across all three markers were combined and represented by a single bar color. Samples are organized by season for each ecozone. RBCL found 38 families with the most abundant family being *Cyperaceae*, TRNL found 18 families with *Salicaceae* most abundant and ITS found 13 families with *Salicaceae* also being the more abundant.

Two metabarcoding markers found a similar number of species, with RBCL identifying 178 species in comparison to 188 species for TRNL while ITS only identified 66 species. At the family level, there was a large difference in diversity with RBCL having over double that of TRNL with 38 and 17 families, respectively, and ITS having the least at 13 families. The muskox's diet was mainly composed of two families, *Cyperaceae* (sedges), and *Salicaceae* (willows). Based on the overall number of reads associated with each family, TRNL had both willows and sedges as its top two families; however, RBCL only had sedges as a dominant family while for ITS the dominant family was willows (Figure 2). Specifically, willows only made up 0.33% of RBCL sequences while sedges were not identified in ITS at all. In addition to willows and sedges, *Fabaceae* (legumes) and *Betulaceae* (birch) were also prominent families based on TRNL sequences. For RBCL, *Rosaceae* (rose), *Betulaceae* (birch), *Ericaceae* (heaths), and *Saxifragaceae* (saxifrages) were dominating families in addition to sedges. Finally, ITS had *Fabaceae* (legumes) and *Rosaceae* (rose) as the dominating families in addition to willows. TRNL and RBCL shared 10 families making up 87.5% of reads in RBCL and 98.8% of reads in TRNL. All of the families found in ITS were also found in both TRNL and RBCL. At a species level, only one species (*Stellaria longipes*) was shared across all three markers, with seven species shared between at least two markers, demonstrating that comparisons among markers should only be performed at higher taxonomic levels.

### 3.3 Relationship between alpha diversity of diet and microbiome

**FIGURE 3:**
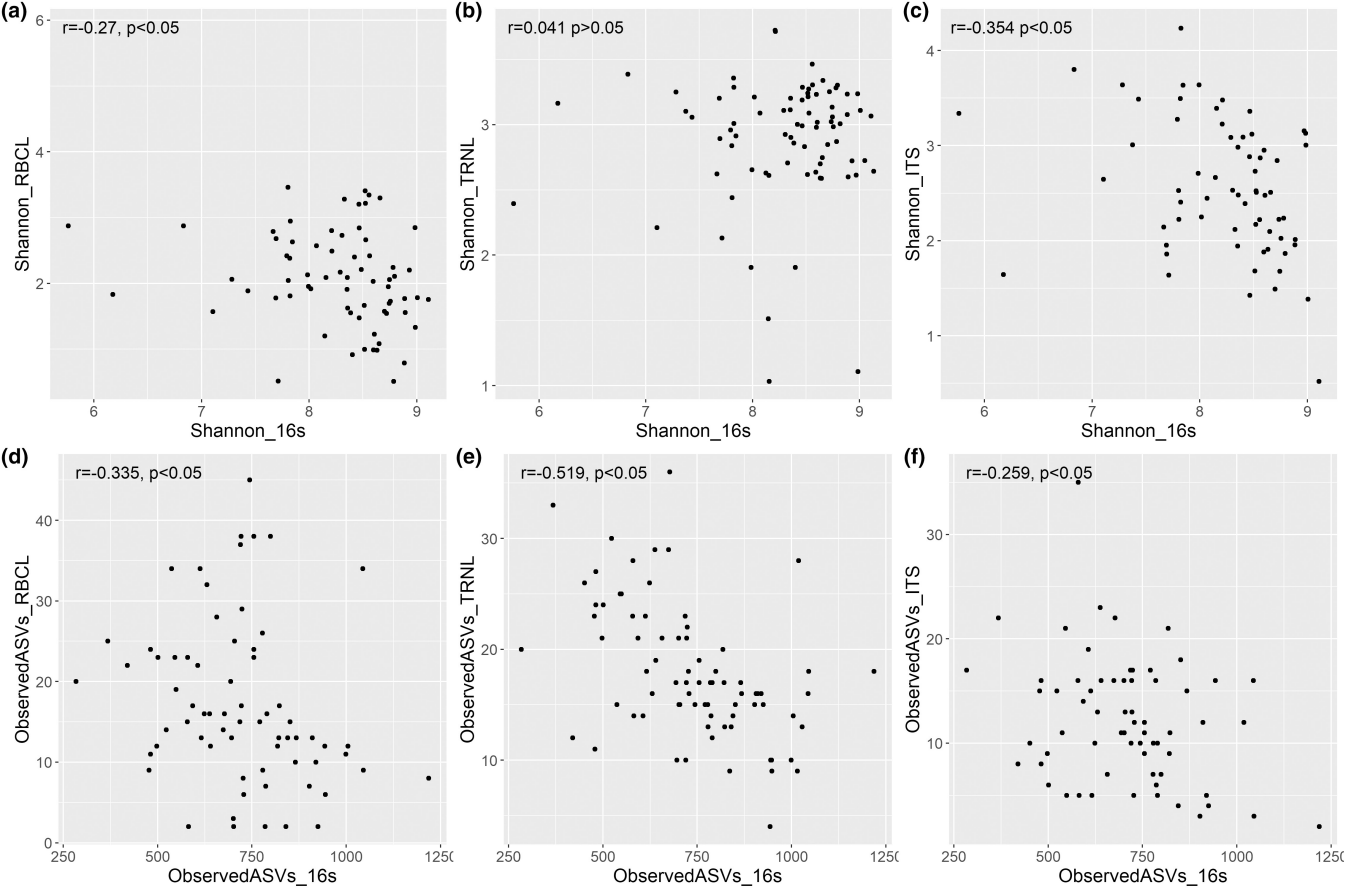
Relationships between alpha diversity metrics of the diet and microbiome metabarcoding from 78 male muskox individuals. (a) RBCL diet Shannon index and 16s microbiome Shannon index. (b) TRNL diet Shannon index and 16s microbiome Shannon index. (c) ITS diet Shannon index and 16s microbiome Shannon index. (d) RBCL diet ASVs, and 16s microbiome ASVs. (e) TRNL diet observed ASVs and 16s microbiome ASVs. (f) ITS diet ASVs, and 16s microbiome ASVs.

We tested the alpha diversity of microbiomes to the corresponding alpha diversity of diets and found a significant, negative correlation between diet and microbiome alpha diversity with *R* ^2^ ranging from −.259 to −.519 (Figure 3). This significant negative relationship was consistent for all markers based on Shannon indices and ASVs with the exception of TRNL and 16s alpha diversity, which was not significant (Figure 3b). We also tested alpha diversity between diet markers to determine whether they were correlated despite not sharing similar species. There was a significant positive correlation between diet alpha diversities of ITS and RBCL *R* ^2^ values at .52 for ASVs and .285 for the Shannon index (Figure S1c,f). However, only observed ASVs were significant between ITS and TRNL with an *R* ^2^ value of .111 and only Shannon index was significant between RBCL and TRNL with an *R* ^2^ value of .335 (Figure S1a,e).

### 3.4 Relationship between beta diversity measures

**FIGURE 4:**
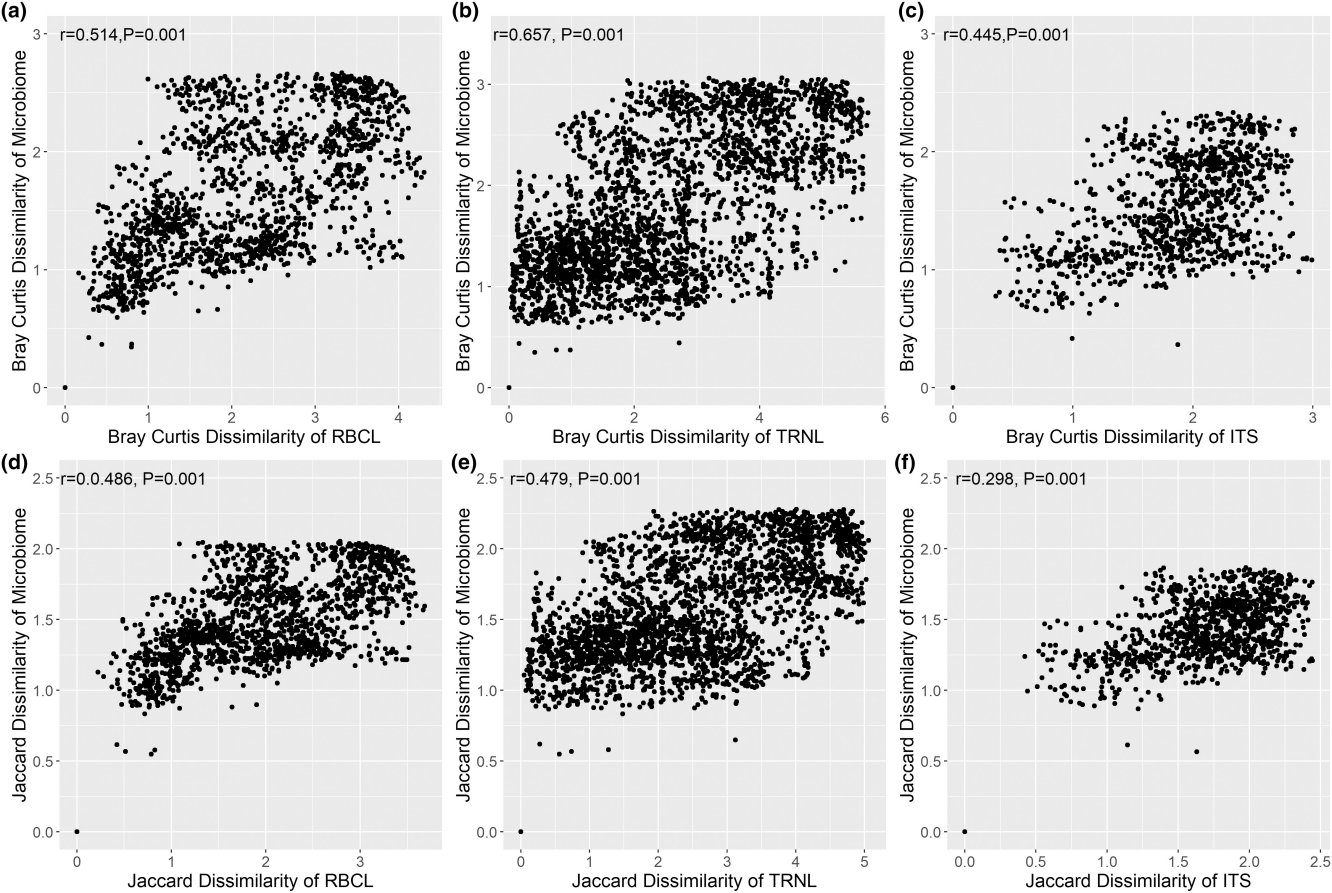
Relationship between beta diversity of (a) RBCL diet Bray–Curtis dissimilarity and 16s microbiome dissimilarity. (b) TRNL diet Bray–Curtis dissimilarity and 16s microbiome Bray–Curtis dissimilarity. (c) ITS diet Bray–Curtis dissimilarity and 16s microbiome Bray–Curtis dissimilarity. (d) RBCL diet Jaccard dissimilarity and 16s microbiome Jaccard dissimilarity. (e) TRNL diet Jaccard dissimilarity and 16s microbiome Jaccard dissimilarity. (f) ITS diet Jaccard dissimilarity and 16s microbiome Jaccard dissimilarity.

We tested beta diversity between microbiomes and diet for correlations, where beta diversity is a measure of the degree to which samples differ from one another. We found significant positive correlations between beta diversity of microbiome and diet, as well as between diet markers (Figure 4). Samples with similar diet were found to have more similar microbiomes where *R* ^2^ ranged from .298 to .657 between diet and microbiome (Figure 4).

## 4 DISCUSSION

We assessed wild muskoxen plant diet composition and diversity via DNA metabarcoding from environmental fecal samples across ecozones and seasons. These data were then compared with observed microbiomes from the same fecal samples to evaluate correlations between diet diversity on that of the microbiome. We observed a lack of concordance between metabarcoding markers employed, with divergent plant species identified, as well as varying plant family abundances within individuals, yet there were consistent general trends found across markers. When comparing diet and microbiome, alpha diversity was negatively correlated, indicating that as diet diversity increases, microbiome diversity decreases. Diet beta diversity and microbiomes were positively correlated, indicating samples with similar diets had more similar microbiomes. These data enhance our understanding of relationships between muskox diet and their microbiome; relationships relevant to assessing the long‐term sustainability of this iconic species in response to climate change and a warming Arctic.

### 4.1 Muskox diet

This study aimed to elucidate muskox diet from three commonly employed plant metabarcoding markers (RBCL, TRNL, and ITS) in an attempt to avoid ascertainment biases that can result from single marker studies and add to the breadth of dietary items identified. While the three markers identified a wide range of species and families, the lack of concordance in the number of families identified and familial abundances indicate clear ascertainment biases among markers and demonstrate the importance of using multiple markers and marker choice for metabarcoding approaches (da Silva et al., 2019; Srivathsan et al., 2015). The lack of concordance observed among the markers could partially be attributed to primer specificity biases, where different primers preferentially bind to specific species due to mismatches, and can change the relative abundances of those families in the reads (da Silva et al., 2019; Deiner et al., 2017). Additionally, universal primer designs for metabarcoding must balance high taxonomic coverage and high taxonomic resolution, which can lead to concordance issues as primer pairs designed to bind to highly conserved sites will likely amplify a larger variety of species but lack the variation to distinguish between said species (da Silva et al., 2019; Hollingsworth, 2011; Moorhouse‐Gann et al., 2018). For example, the use of ITS has been criticized due to its lower universality in comparison to other markers. ITS, however, has a higher power to discriminate where TRNL has been found to have ambiguity problems at the genus and species level (da Silva et al., 2019; Moorhouse‐Gann et al., 2018). The level of sample degradation can also affect the concordance of metabarcoding results where markers with shorter amplicon sizes better amplify degraded samples but have lower power to distinguish between species (Coghlan et al., 2021; Mallott et al., 2018; Ruppert et al., 2019). To illustrate this point, Mallott et al. (2018) used metabarcoding of the TRNL (~150 bp) and RBCL (~500 bp) markers to study diets of capuchin monkeys. They found that TRNL produced a higher number of sequences, more unique sequences, and that more sequences were identified to the order or family level, while RBCL had more reads that were identifiable to species level (Mallott et al., 2018). Similarly, we amplified and retained more sequences with the TRNL marker in our study, while the RBCL marker identified more families. A lack of reference reads across the databases could also partly account for the lack of concordance and overlapping species between markers. To assess this possibility, we searched the databases for sequences of species that were highly represented by one marker and missing in the others. Representative sequences were found across all markers indicating that this was not the cause of minimal overlapping species.

Despite differences in diversity and abundance of plant families between markers in this study, our results do align to previous observations that found willow (*Salicaceae*) and sedge (*Cyperaceae*) families comprise up to 95% of the muskox's diet using both metabarcoding and histological methods (Ihl & Klein, 2001; Larter & Nagy, 1997, 2004; Schmidt et al., 2018). We found also found legumes (*Fabacea*), rose (*Rosaceae*), heath (*Ericaceae*), birch (*Betulaceae*), and saxifrage (*Saxifragacea*) families to be large contributors outside of willows and sedges, though no marker found all these families in high abundance. Overall, with both sedges and willows identified as the major contributors to diet, TRNL provided results most consistent with previous histological studies that sampled from similar regions and seasons but lacked family‐level diversity identified by RBCL (Ihl & Klein, 2001; Larter & Nagy, 1997, 2004). While histological studies (Ihl & Klein, 2001; Larter & Nagy, 1997, 2004) have sampled muskoxen across multiple seasons and geographic regions, improved taxonomic resolution and sensitivity in detecting low‐abundance plant families were observed with metabarcoding tools. Previous metabarcoding of muskox diet performed by Schmidt et al. (2018), similarly found that muskoxen ate mainly shrubs, forbs, and graminoids, though they did not find a high abundance of sedges. While findings from Schmidt et al. (2018) differ from the results found herein, all 20 samples from their study were collected in winter from a 7‐km radius area in Greenland. As such, data from Schmidt et al. (2018) would not be expected to encompass the variation of plants eaten by muskoxen across multiple seasons and ecozones in the Canadian Arctic (Schmidt et al., 2018). Although the sampling scheme herein is also limited by the distribution of samples over season and ecozones, results from the current study may provide a more complete picture of muskox diet diversity in the Canadian Arctic. While metabarcoding has improved the ability to detect low‐abundance plants, the use of fecal samples to assess diet may be biased against species that are highly digestible. This might result in an artificially high abundance of fibrous plants like willows and sedges as they are harder to digest. Overall, despite a lack of consensus between these three markers regarding specific diet composition, there were consistent trends identified from the metabarcoding data from this study in relation to diet diversity and the microbiome.

### 4.2 Diet and microbiome diversity correlations

Samples used in this study were the same as those used by Bird et al. (2019), providing an opportunity to directly compare microbiome diversity to diet diversity in wild muskoxen. We compared both beta diversity (diversity between samples) and alpha diversity (diversity within samples) of muskox diets and microbiomes. We found significant positive correlations between beta diversities of microbiomes and diet, where individuals with more similar diets had more similar microbiomes. When comparing alpha diversities, however, there was a significant negative correlation between these data types. This was unexpected, as a more diverse diet is thought to introduce more diverse microbiota and nutrients to the gut and therefore require, or lead to, higher microbiome diversity (Bolnick et al., 2014). Few studies have yielded similar negative correlations between microbiome and diet diversity, such as Bolnick et al. (2014) who found a negative correlation of alpha diversity in wild and laboratory‐raised freshwater fish, where generalist fish, expected to have more diverse diets, had lower microbiome diversities. Li et al. (2016), compared the microbiome and diet of pika and found that while there was a positive correlation of beta diversity, alpha diversity was not correlated in this species. Both Bolnick et al. (2014) and Li et al. (2016) suggest several hypotheses as to how more diverse diets could lead to reduced gut microbiome diversity. First, certain foods may contain chemical inhibitors that reduce or eliminate certain bacteria, therefore the more diverse the diet, the more inhibitors they would ingest. It was also hypothesized that diet influences host health and immunity, which affects an individual's ability to respond to novel microbial species or microbial infections (Bolnick et al., 2014; Li et al., 2016). Finally, these authors proposed that some dominant species of microbes function or survive better on a wider range of nutrients derived from more diverse diets, as opposed to more rare microbes that have more specific nutrient needs (Bolnick et al., 2014; Li et al., 2016). While previous studies provide general explanations for unusual microbiome and diet correlations, there may be factors specific to muskoxen that contribute to the trends observed in the current study.

As stated, the muskox diet consists primarily of willows and sedges (up to 95%) (Ihl & Klein, 2001; Larter & Nagy, 1997, 2004; Mosbacher, Michelsen, et al., 2016), suggesting this species is selective in their diet, if only at the familial level. Similarly, Schmidt et al. (2018) found that when food availability was low, the diets of muskoxen, rock ptarmigans, and Arctic hares did not overlap, and muskoxen continued to consume the lowest diversity diet (Schmidt et al., 2018). This might suggest microbiomes of muskoxen are adapted primarily to these two plant families, rather than the presence of other families in low abundance. Therefore, the muskox microbiome may be more influenced by the relative abundance of the ingested plant families, rather than the diet diversity in terms of plant families present. Additionally, sedges and willows are high‐fiber plants, thus muskox microbiome compositions may be adapted to the digestion of fiber specifically. Bird et al. (2019) found the major bacterial orders in muskox samples were *Firmicutes* and *Bacteroidetes*, species known to help fiber and carbohydrate digestion. However, if willows and sedges were key families affecting microbiome diversity, we would expect to see similar results to that of Li et al. (2016), where beta diversity between microbiome and diet was correlated, but alpha diversity was not, as these families were consistently found to make up a large percentage of the muskox diet. While the presence of these plant families may be consistent within the muskox diet, the quality of the willows and sedges can vary across ecozones, seasons, and years (Larter & Nagy, 2001), which we speculate may also affect the muskox microbiome.

Another factor that could impact the relationships between diet and microbiome of muskoxen is the amount of food eaten. Metabarcoding methods have the potential to be more sensitive and accurate in their detection of plant species, but they are unable to measure the volume of food eaten and how this impacts a species' microbiome. Food intake is known to affect the concentration of fermentation acids, ruminal pH, and bacterial conditions in muskoxen that in turn may limit microbial communities and suppress more active microbes (Barboza et al., 2006). In species that have high‐fiber diets, a slow passage rate allows for increased food breakdown and nutrient extraction (Barboza et al., 2006). However, hyperphagia results in an increased or fast passage of food and therefore may be more detrimental to digestion when it occurs in muskoxen (Barboza et al., 2006). Decreased food intake in winter has been documented in captive muskoxen where the same food is available throughout the seasons, indicating further that low food intake over winter is beneficial/adaptive for muskoxen (Adamczewski, Flood, et al., 1994). Decreases in food volume also lower the number of bacteria in their rumen overall, rather than just the number of species present (Barboza et al., 2006; Crater & Barboza, 2007). This helps maintain ideal digestion rates for decreased food intake but also makes it easier to detect lower quantity bacteria species that may have previously been outcompeted and consequently, increases measures of microbiome diversity.

### 4.3 Muskox diet, microbiome, and Arctic warming

Arctic warming is changing the diversity and composition of Arctic plant communities (Mekonnen et al., 2021; Mod & Luoto, 2016) and specifically, results in shrubification, a rise in shrub abundance via increased growth and progressive shrub lines (Mekonnen et al., 2021; Mod & Luoto, 2016). As shrubification continues, increased competition for native Arctic plant species is predicted to reduce tundra plant biodiversity (Mekonnen et al., 2021; Mod & Luoto, 2016). This in turn could impact the survival and biodiversity of Arctic herbivores that fall within dietary niches that consist of native Arctic plants (Mekonnen et al., 2021; Mod & Luoto, 2016; Mosbacher, Kristensen, et al., 2016; Schmidt et al., 2018). Some examples of shrub families increasing in abundance include willows, consistently found to play an important role in muskox diet, and birch, which muskoxen are thought to typically avoid but were found in relatively high abundances herein. Shrubification may then have the potential to aid muskox populations by providing additional resources (Mekonnen et al., 2021; Mod & Luoto, 2016; Mosbacher, Kristensen, et al., 2016). In Greenland, muskox grazing reduced the extent of shrubification occurring, diminishing the effects of Arctic warming on Arctic vegetation and increasing plant biodiversity in grazed areas (Mosbacher, Kristensen, et al., 2016; Post et al., 2021; Post & Pedersen, 2008). While an increased availability of shrubs like willows and birch may be promising, increasing their relative abundance in the muskox diet may not be ideal, as nutrient content affects diet selection and their microbiome. Lawler and White (1997) investigated the effects of browse, like willows and birch, to muskox diet and found muskoxen rejected feed containing over 60% browse, while feed with over 40% browse resulted in a postingestion energy loss (Lawler & White, 1997). These results were thought to be linked to energy required for the detoxification of poisonous secondary compounds like tannins found in leaves and twigs of willows (Lawler & White, 1997). Further, these findings indicate that though muskoxen could benefit from an abundance of shrubs in the Arctic, they require access to graminoid species as well. The loss of other plant species, particularly graminoid species from shrubification, could eventually result in diets that are more harmful and cost more energy to muskoxen.

Arctic warming not only affects Arctic plant species diversity but the phenology of local vegetation. Availability and reliability of vegetation play a role in muskox demographics with earlier green‐up times associated with increased population sizes and plant consumption (Eikelenboom et al., 2021; Koltz et al., 2022; Post et al., 2019). Muskoxen are capital breeders and are expected to be relatively insensitive to changing plant phenology as their breeding is reliant on their body reserves from the past year, rather than resources obtained during the breeding period (Gustine et al., 2010; Kerby & Post, 2013). As green‐up times occur earlier, there would be an expected increase in trophic match, where longer growing seasons are associated with increased muskox abundance (Kerby & Post, 2013; Koltz et al., 2022; Post et al., 2019). Additionally, with advancing green‐up, there is improved calf recruitment in muskoxen, as earlier and more abundant food lowers the cost of lactation on the mothers and calves have higher quality forage when they transition to grazing (Eikelenboom et al., 2021). However, increases in muskox populations from early green‐ups are expected to eventually stabilize or decline as winters become more wet and snow depths increase (Eikelenboom et al., 2021). High snow depth and coverage limit muskox accessibility to willows and can result in periods of starvation and weight loss (Desforges et al., 2021; Mosbacher, Kristensen, et al., 2016). Body stores that would normally aid calf growth then become necessary for the survival of cows, leading to poor calf recruitment (Mosbacher, Kristensen, et al., 2016). Thus, while speculative, changes to vegetation diversity and phenology may benefit muskoxen in the short term, but as effects continue, muskoxen populations are at risk of decline in the long term.

## 5 CONCLUSION

Metabarcoding markers employed in this study did not provide fully concordant diversity and composition results, yet revealed willows and sedges as major dietary contributors for muskoxen; a finding consistent with previous metabarcoding and microhistology studies (Ihl & Klein, 2001; Larter & Nagy, 1997, 2004; Schmidt et al., 2018). Comparisons of diet and microbiome diversity found similar diets had more similar microbiomes; however, alpha diversity between microbiome and diet was negatively correlated. The negative correlation was unexpected and may be due to muskox adaptation to high‐fiber diet when access to vegetation is limited, as it is in non‐summer months in the high Arctic. Marker inconsistencies undermined the capacity to directly measure how diet composition impacted microbiome and future studies with additional markers could help clarify these patterns. In particular, the addition of markers that can amplify lichen species, which muskoxen are known to ingest, could provide insight into how these organisms contribute to microbiome diversity. Testing the efficiency of the markers binding to the most abundant plant families could also be performed in future to strengthen diet metabarcoding analyses and assess marker reliability (Buglione et al., 2018). Further, adding more individuals to provide even sampling across ecozones and seasons would allow for analyses of spatial and temporal differences in the muskox diet. With extremely low levels of genetic variation across muskox populations, there are concerns that muskoxen cannot rapidly adapt to the environmental changes brought on by climate change (Cuyler et al., 2019; Gunn & Forchhammer, 2022; Hansen et al., 2018; Prewer et al., 2019). In other systems of long‐lived, low genetic diversity species microbiome plasticity has been postulated as a mechanism to rapidly adapt to climate change and thus may play a role in long‐term muskox sustainability (Bird et al., 2019; Cuyler et al., 2019; Gunn & Forchhammer, 2022; Kutz et al., 2015). Overall, the data from this study provides insight into the relationship between muskox diet and microbiome, which will further our understanding of how muskoxen can adjust to shifting vegetation due to rapid Arctic warming.

## AUTHOR CONTRIBUTIONS

**Erin Prewer:** Conceptualization (equal); data curation (lead); formal analysis (lead); investigation (lead); methodology (lead); visualization (lead); writing – original draft (lead); writing – review and editing (equal). **Sibelle T. Vilaca:** Conceptualization (equal); formal analysis (supporting); investigation (supporting); methodology (supporting); supervision (supporting); writing – review and editing (equal). **Samantha Bird:** Conceptualization (equal); data curation (supporting); formal analysis (supporting); investigation (supporting); methodology (supporting); writing – review and editing (equal). **Susan Kutz:** Data curation (supporting); funding acquisition (equal); resources (equal); writing – review and editing (equal). **Lisa‐Marie Leclerc:** Data curation (supporting); resources (equal); writing – review and editing (equal). **Christopher J. Kyle:** Conceptualization (equal); funding acquisition (equal); resources (equal); supervision (lead); writing – original draft (supporting); writing – review and editing (equal).

## BENEFIT SHARING STATEMENT

This research was possible due to the collaboration, cooperation, and contribution among the scientists, government agencies and community hunters included as co‐authors or described in acknowledgements. This collaboration provided genetic resources, including fecal samples, for the microbiome and diet metabarcoding. This research addresses a priority concern regarding the conservation of muskoxen relative to shifting food resources as a result of climate change. The data and results described in this study have been shared with the broader scientific community via public databases as described above.

## Supporting information


Figure S1.

## Data Availability

Raw sequence reads for diet metabarcoding and ASV tables are available from Dryad (https://doi.org/10.5061/dryad.h9w0vt4n3). Raw sequence reads for microbiome metabarcoding and ASV tables are available from Dryad (https://doi.org/10.5061/dryad.fj6q573q2).
