# Supplementary material for: Metabarcoding of fecal pellets in wild muskox populations reveals negative relationships between microbiome and diet alpha diversity
Source: Ecol Evol. 2023 Jun 13;13(6):e10192. doi: 10.1002/ece3.10192 (PMC10261903; doi:10.1002/ece3.10192)
Supplement: Supplementary file 1 — Figure S1. [file ECE3-13-e10192-s001.pdf]

MOLECULAR ECOLOGY

Supplemental Information for:

Metabarcoding of fecal pellets in wild muskox populations reveals negative relationships between microbiome and diet alpha diversity.

Erin Prewer, Sibelle T. Vilaça, Samantha Bird, Susan Kutz, Lisa-Marie Leclerc, Christopher J. Kyle

Table of Contents:

**Supplemental Figure 2**	Page 1

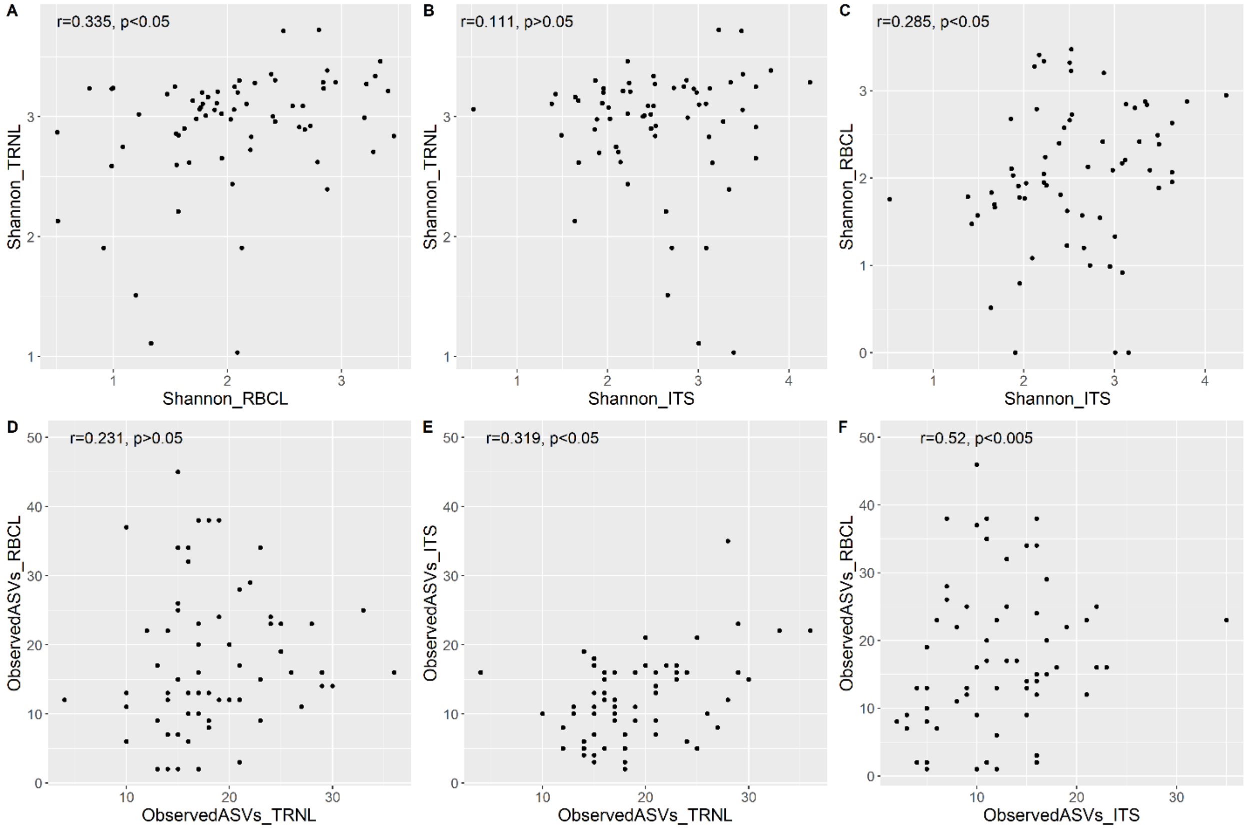

Supplemental Figure 1. Relationships between alpha diversity of (A) RBCL and TRNL diet Shannon index. (B) TRNL and ITS diet shannon index. (C) RBCL and ITS diet Shannon index. (D) RBCL and TRNL diet observed ASVs. (E) ITS and TRNL diet observed ASVs. (F) RBCL and ITS diet observed ASVs
